# 1/*f*-noise-free optical sensing with an integrated heterodyne interferometer

**DOI:** 10.1038/s41467-021-22271-4

**Published:** 2021-03-30

**Authors:** Ming Jin, Shui-Jing Tang, Jin-Hui Chen, Xiao-Chong Yu, Haowen Shu, Yuansheng Tao, Antony K. Chen, Qihuang Gong, Xingjun Wang, Yun-Feng Xiao

**Affiliations:** 1 State Key Laboratory of Advanced Optical Communications System and Networks, Department of Electronics, School of Electronics Engineering and Computer Science, Peking University, Beijing, 100871 China; 2 State Key Laboratory for Artificial Microstructure and Mesoscopic Physics, School of Physics, Peking University, Beijing, 100871 China; 3 Department of Biomedical Engineering, College of Engineering, Peking University, Beijing, 100871 China; 4 Frontiers Science Center for Nano-optoelectronics, Peking University, Beijing, 100871 China; 5 Collaborative Innovation Center of Extreme Optics, Shanxi University, Taiyuan, 030006 China; 6 Peking University Yangtze Delta Institute of Optoelectronics, Nantong, 226010 China

**Keywords:** Electrical and electronic engineering, Optical sensors, Imaging and sensing, Biophotonics

## Abstract

Optical evanescent sensors can non-invasively detect unlabeled nanoscale objects in real time with unprecedented sensitivity, enabling a variety of advances in fundamental physics and biological applications. However, the intrinsic low-frequency noise therein with an approximately 1/*f*-shaped spectral density imposes an ultimate detection limit for monitoring many paramount processes, such as antigen-antibody reactions, cell motions and DNA hybridizations. Here, we propose and demonstrate a 1/*f*-noise-free optical sensor through an up-converted detection system. Experimentally, in a CMOS-compatible heterodyne interferometer, the sampling noise amplitude is suppressed by two orders of magnitude. It pushes the label-free single-nanoparticle detection limit down to the attogram level without exploiting cavity resonances, plasmonic effects, or surface charges on the analytes. Single polystyrene nanobeads and HIV-1 virus-like particles are detected as a proof-of-concept demonstration for airborne biosensing. Based on integrated waveguide arrays, our devices hold great potentials for multiplexed and rapid sensing of diverse viruses or molecules.

## Abstract (web summary)

Suppressing 1/f-shaped low-frequency noise is critical but fundamentally challenging to both electrical and optical transducers. Here, the authors demonstrate a 1/f-noise-free optical sensor with integrated CMOS-compatible heterodyne interferometer and an upconversion amplifying technique, which suppresses the noise by two orders of magnitude.

## Introduction

Low-frequency noise with the spectral density *S*(*f*) = 1/*f*^*γ*^ (where *f* is the frequency, and *γ* commonly ranges from 0.5 to 1.5), well known as 1/*f* noise, flicker noise, or excess noise, has been a ubiquitous phenomenon in both electrical and optical systems^1–4^. In general, the 1/*f* noise originates from the carrier instability inside photoelectric materials^4,5^ or the current flowing through electrical circuits^6,7^. Effectively suppressing the low-frequency noise is critical but fundamentally challenging to both electrical and optical transducers, especially to the ultrahigh-sensitivity detection and analysis. For example, in optical evanescent sensors based on resonant structures^8–10^ and nanowaveguides^11–15^, 1/*f* noise poses serious difficulties for resolving dynamic signals, such as biomolecule motions, binding, and trapping which are typically charaterized by hertz to kilohertz frequencies^16–22^. To date, while much effort has been devoted to suppressing the optical fluctuations in evanescent sensors, such as self-reference mode splitting^23–26^, frequency tracking^27–30^, and lock-in amplifying^14,19^, the 1/*f* noise suppression in optical sensors has never been explored.

Here, we propose an ultralow-noise optical sensing scheme, which can effectively suppress the 1/*f* noise, via an integrated heterodyne interferometer and an up-conversion amplifying technique. In experiment, the sampling noise is reduced by two orders of magnitude, compared with conventional lock-in amplifying method that focuses on suppressing optical fluctuations^14,19^. The ultralow-noise sensor is applied to detect single polystyrene and HIV-1 virus-like particles with the detection limit down to a few attograms. The 1/*f*-noise-free sensing scheme, combined with conventional enhancement mechanisms based on plasmonic nanostructures^15,17,31^ or surface charges on the analytes^19,30^, can provide a powerful way towards studying single-molecule dynamics in fundamental biophysics research and practical biological applications.

## Results

### 1/*f*-noise-free scheme

**Fig. 1:**
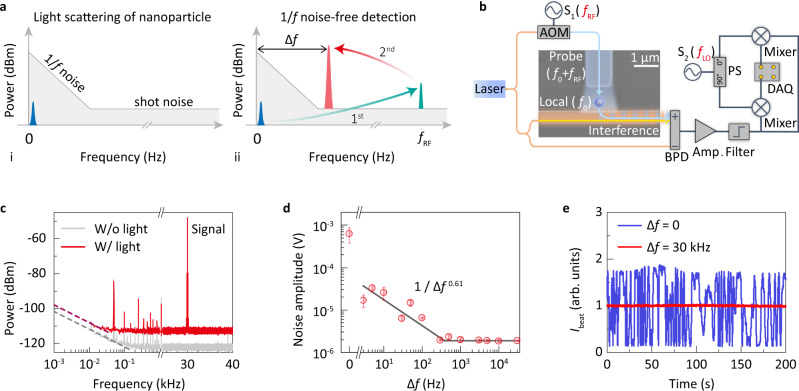
1/*f*-noise-free optical sensor. **a** A low-frequency light scattering signal induced by a nanoparticle buried in noise background (i), and the principle of 1/*f*-noise-free detection (ii). Blue peak: original light scattering signal; green peak: the first amplified signal after optical up-conversion process; red peak: the extracted signal after electrical amplification and down-conversion processes. **b** Experimental set-up. BPD balanced photodetector, S_1_ and S_2_ signal generators with *f*_RF_ = 80.15 MHz and *f*_LO_ = 80.18 MHz, respectively; AOM acousto-optic modulator, Amp. radio-frequency amplifier, PS 90^∘^ power splitter, DAQ data acquisition system. **c** Power spectra of ${A}_{{\rm{beat}}}^{{\rm{I}}}$ at low frequency region when both probe and local light are off (gray curve) or on (red curve). The low-frequency noise floor is fitted by 1/*f* (dashed curves). **d** The sampling noise amplitude versus bias frequency Δ*f*. Red circles: experimental data; black curve: 1/*f*^0.61^ fitting below the corner frequency. Error bars represent the standard deviation of repeated measurements. **e** Beat intensity *I*_beat_ for the conventional lock-in amplifying scheme (Δ*f* = 0, blue curve) and the 1/*f*-noise-free regime (Δ*f* = 30 kHz, red curve).

The 1/*f*-noise-free scheme is presented in Fig. 1a. We consider a weak photoelectric signal within sub-kilohertz frequency, which falls into the low-frequency 1/*f* noise band and could not be resolved through a conventional sensing method (Fig. 1a–i). To extract this tiny signal, first, the probe light carrying the sensing signal is shifted by *f*_RF_ and optically amplified through a heterodyne interferometry. To greatly reduce the effect of 1/*f* noise, the beat frequency *f*_RF_ is typically chosen to be tens of megahertz. Second, the beat-frequency photoelectric response is electrically boosted and down converted to a bias frequency Δ*f*, considering a finite sampling rate during the long-term observation. For a conventional data acquisition card, the sampling frequency *f*_sampling_ is approximately hundreds of kilohertz. The Δ*f* should fall into *f*_sampling_ but still be beyond the low-frequency 1/*f* noise band. In this way, the low-frequency signals can be detected with greatly suppressed noise and high-gain amplification in the electric domain.

The above scheme is applied to detect single nano-objects with the experimental set-up shown in Fig. 1b. The heterodyne interferometer, fabricated via a commercial silicon photonics foundry process, consists of a local waveguide and a probe waveguide. They are perpendicularly patterned with a gap serving as the joint sensing area. Since very little of the probe field is collected into local waveguide in the absence of nanoparticles, this dark-field waveguide configuration enables minimal background noise introduced by the probe light (Fig. 1b and Supplementary Fig. 1)^19,32^. The traveling light in the local (probe) waveguide has the frequency *f*_0_ (blued shifted by *f*_RF_ = 80.15 MHz, *f*_0_ + *f*_RF_).

When single nanoparticles are deposited into the joint area, due to the elastic scattering, a part of the probe light is collected by the local waveguide via evanescent-wave coupling, which interferes with the local light (Fig. 1b). The generated beat-frequency envelop together with a reference light is subsequently detected by a balanced photodetector where the laser noise can be significantly suppressed^21^. In this scheme, the scattered signal is boosted by a dual amplification strategy. First, it is optically amplified in dark-field heterodyne configuration by a strong local field, and the enhancement factor reads $2\sqrt{{P}_{{\rm{local}}}/{P}_{{\rm{probe}}}}$ with *P*_local_ and *P*_probe_ being the power of local and probe light, respectively. The beat-frequency signal is further boosted by ~ 500 times using a radio-amplifier. After the cross-phase down conversion with the frequency *f*_LO_ = 80.18 MHz, two channel electrical signals (${A}_{{\rm{beat}}}^{{\rm{I}}}$ and ${A}_{{\rm{beat}}}^{{\rm{Q}}}$) are recorded at the bias frequency Δ*f* = *f*_LO_ − *f*_RF_ = 30 kHz without distortion, which is also beyond the 1/*f* noise band. The beat intensity *I*_beat_ = ${({A}_{{\rm{beat}}}^{{\rm{I}}})}^{2}+{({A}_{{\rm{beat}}}^{{\rm{Q}}})}^{2}$ scales linearly with the scattering efficiency of the nanoparticle (Supplementary Note 1).

### Noise analysis

To study the noise performance in the low-frequency band, the power spectra of ${A}_{{\rm{beat}}}^{{\rm{I}}}$ are obtained with or without light input as shown in Fig. 1c. In both cases, the significant electronic noise with the 1/*f* spectral shape emerges below ~100 Hz. In addition, a mass of spikes can be found with frequencies within 1 kHz that are introduced by the electrical elements. To eliminate these disturbances during extracting process, the beat-frequency signal is thus sampled at a bias frequency Δ*f* far away from the low-frequency noise band (red curve in Fig. 1c). Furthermore, the noise amplitude of ${A}_{{\rm{beat}}}^{{\rm{I}}}$ is plotted as a function of the bias frequency Δ*f* as shown in Fig. 1d, and it is fitted by 1/(Δ*f*)^0.61^ below the corner frequency of ~300 Hz. It is found that the sampling noise amplitude is suppressed by two orders of magnitude than that of the conventional lock-in amplifying method (1.87 × 10^−6^ V at Δ*f* = 30 kHz versus 6.22 × 10^−4^ V at Δ*f* = 0). Here, the sampling noise amplitude is calculated from the standard deviation of the cross-correlation $\frac{2}{T}\mathop{\int}\nolimits_{-T/2}^{T/2}{A}_{{\rm{beat}}}^{{\rm{I}}}(t+\tau )h(\tau ){\mathrm{d}}\tau$ between the signum function *h*(*t*) = sgn(*t*) and the signals ${A}_{{\rm{beat}}}^{{\rm{I}}}$ in the duration time *T* = 1 s (Supplementary Note 2). With such a 1/*f*-noise-free extraction method, the corresponding real-time signals *I*_beat_ in Fig. 1e show a significant suppression of 12.3 dB on the *I*_beat_ fluctuations.

### Sensing characterization with a near-field nanotip

**Fig. 2:**
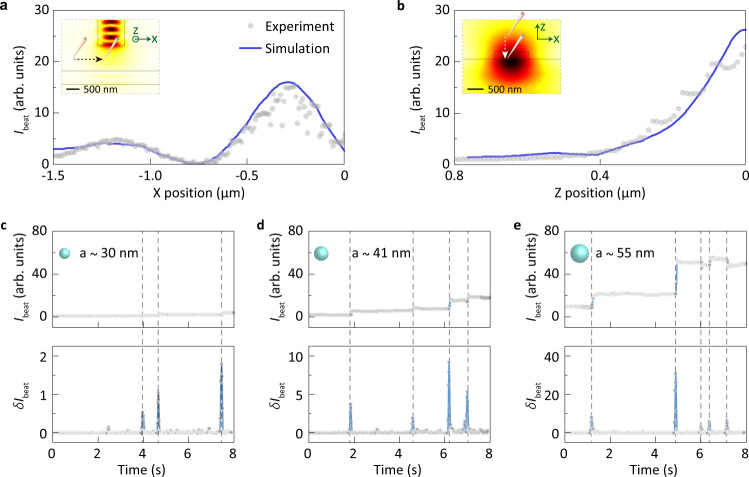
Characterization of sensing performance. **a**–**b** Beat intensity *I*_beat_ when a silica nanotip with the radius of 200 nm is horizontally and vertically scanned for 1.5 μm (**a**) and 0.8 μm (**b**), respectively. Insets, field distributions of fundamental TE mode of a waveguide and near-field scanning range of the nanotip. **c**–**e** Beat intensity *I*_beat_ (top) and the corresponding *δI*_beat_ (bottom) when single polystyrene nanobeads with the radii of 30 nm (**c**), 41 nm (**d**), and 55 nm (**e**) are deposited at the joint sensing region.

The on-chip waveguide sensor enables quantitative characterization of its sensing performance through a near-field nanotip as shown in Fig. 2a, b. A silica nanotip with a radius of 200 nm is applied as a controllable nano-object to observe the local topography of the joint sensing region (Methods). Experimentally, the nanotip is horizontally and vertically scanned in the range of 1.5 μm and 0.8 μm as shown in the insets of Fig. 2a, b, respectively. The beat intensity *I*_beat_ as a function of nanotip position is in good agreement with the simulation results. In the three-dimensional finite element method (3D FEM) simulation, the power of probe light collected by the local waveguide *P*_col_ is calculated as a function of the particle position (200-nm-radius silica nanosphere). As expected, the *I*_beat_ increases as the nanotip approaches the joint sensing area, in which the signal-noise ratio (SNR) as high as 2 × 10^3^ is observed. Note that the signal *I*_beat_ reaches its maximum when the nanotip is about 300 nm away from the center of probe waveguide in Fig. 2a, which shows a directional scattering behavior (Supplementary Note 3)^33^.

### Real-time detection of single nanoparticles

The waveguide sensor is then tested by the standard polystyrene nanobeads. Experimentally, the nanobeads (30, 41, and 55 nm in radius) are blown onto the joint sensing area using a glass nozzle via a syringe pump (Methods). The binding events of nanoparticles are clearly recognized by the discrete increasing steps in real-time beat signal *I*_beat_ as shown in Fig. 2c–e. As expected, the height of the discrete steps in $\delta {I}_{\text{beat}}=\frac{2}{T}\mathop{\int}\nolimits_{-T/2}^{T/2}{I}_{{\rm{beat}}}(t+\tau )h(\tau ){\mathrm{d}}\tau$ increases with the particle size. A few decreasing steps are also observed, which is due to the nanoparticles blown away by the air flow from the glass nozzle or the interference effects induced by multiple nanoparticles^12,13^. For the 30-nm-radius nanobeads, discrete steps of beat intensity *I*_beat_ with the SNR as high as 14.5 dB are observed, while a SNR below 0 dB is observed at the same time with the conventional waveguide sensor through directly monitoring the transmission loss^12,13^ (Supplementary Fig. 4). The enhanced SNR is contributed by the dual signal amplification strategy and the 1/*f* noise suppression.

**Fig. 3:**
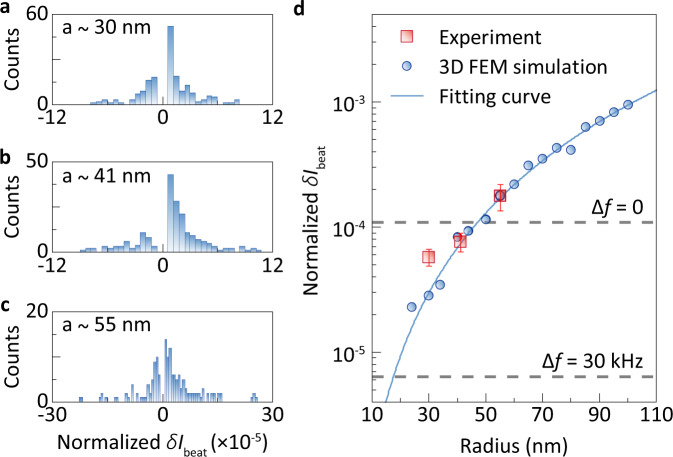
Analysis of detection limit. **a**–**c** Histograms of normalized beat intensity change *δI*_beat_ for standard polystyrene nanobeads with radii of 30 nm (**a**), 41 nm (**b**), and 55 nm (**c**). **d** Normalized *δI*_beat_ induced by standard polystyrene nanobeads versus radius. Blue dots are the scattering efficiencies of the nanoparticle obtained by 3D FEM simulation. The fitting curve shows *a*^2.85^ dependence. The dashed lines are the noise levels for Δ*f* = 0 and 30 kHz, respectively. Error bars represent the standard deviation.

To estimate the detection limit, the statistical steps in the interval [*δI*_beat_ − *σ*/2, *δI*_beat_ + *σ*/2] with *σ* = 3 × 10^−6^ in Fig. 3a–c, originated from the particles deposited closely to the joint sensing area, are selected to analyze the size-dependent behavior. The mean values and standard deviations of the selected *δI*_beat_ are plotted in Fig. 3d, which are in good agreement with the simulation results. In the 3D FEM simulation, the power of probe light collected by the local waveguide *P*_col_ is calculated as a function of particle size when the scatterer is placed in the joint sensing area. Since the beat intensity is proportional to the collected power of probe light *δI*_beat_ = *rP*_col_ (Supplementary Note 1), the equipment-dependent coefficient *r* is obtained according to the simulated *P*_col_ and the corresponding measured *δI*_beat_ induced by 55-nm-radius polystyrene nanobeads. The noise levels derived from 3*σ* of *I*_beat_ is 6.64 × 10^−6^ (1.12 × 10^−4^) at Δ*f* = 30 kHz (Δ*f* = 0), where *σ* is the standard deviation. According to the fitting curve of simulated results in Fig. 3d, the detection limit is 17.5 nm in radius for Δ*f* = 30 kHz, while it is 47.3 nm for Δ*f* = 0. This indicates that the ultrasensitive and low-noise sensor enables detection of single nanoparticles with the mass down to a few attograms (10^−18^ g).

**Fig. 4:**
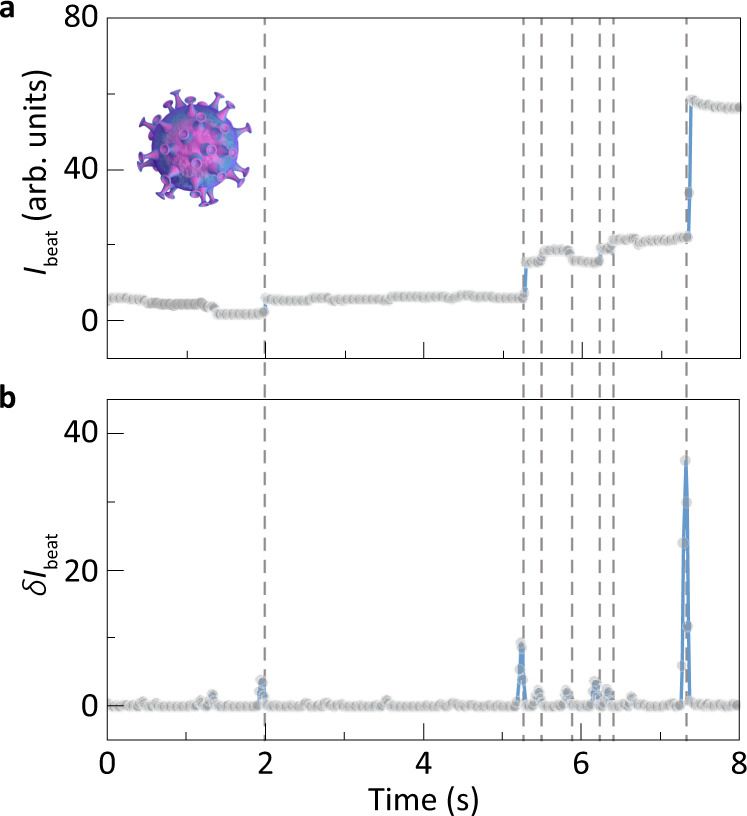
Detection of single virus-like particles. **a**–**b** *I*_beat_ (**a**) and the corresponding *δI*_beat_ (**b**) when HIV-1 virus-like particles are deposited at the joint sensing region.

Detecting and characterizing viruses in aerosol are of paramount importance for disease control and diagnosis^34^. Here, as a proof-of-concept demonstration for airborne biosensing, we applied the low-noise sensor to detect single viruses. The virus-like particles (VLPs) assembled from living cells expressing the human immunodeficiency virus (HIV) gag protein (Methods) are detected. Figure 4a presents clear discrete steps in the beat intensity *I*_beat_ when single particles are blown onto the sensing area. In order to make a clearer comparison between the height of *I*_beat_ steps versus the noise level, the absolute value of *δI*_beat_ calculated through cross-correlation is shown in Fig. 4b. The maximum SNR as high as 20 dB is obtained for the HIV-1 virus-like particles, suggesting our sensor has the ability to detect smaller viruses. Furthermore, the *δI*_beat_ of detected VLPs in an ensemble measurement is shown in Supplementary Fig. 5. Considering the difference in refractive index between virus and polystyrene nanobeads, the estimated radius of VLPs is about 52.1 ± 1.9 nm, which is close to the expected size of an HIV-1 VLP (~50 nm in radius).

### Multiplexed and rapid sensing

**Fig. 5:**
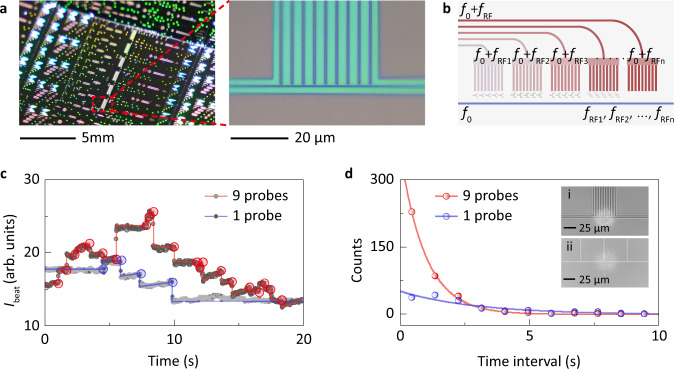
Waveguide array on a chip for rapid and multiplexed sensing. **a** Optical images of the waveguide sensor on a chip. **b** Proposed scheme for the radio-frequency multiplexing. **c** Beat intensity *I*_beat_ when single polystyrene nanobeads are deposited onto the sensing area of nine (red curve) and one (blue curve) probe-waveguide configurations, respectively. **d** Statistical distributions of time intervals between two sequential nanobead-binding events fitted by the exponential curve. Insets, nine (i) and one (ii) probe-waveguide configurations after nanoparticle deposition.

The ultralow-noise sensor is realized with the high-density integrated waveguide arrays via a commercial silicon photonics foundry process (Fig. 5a). This feature enables to explore multiplexed and rapid sensing.

A radio-frequency multiplexing concept is proposed as shown in Fig. 5b. The lights (*f*_0_) shifted by the radio frequencies (*f*_RF1_, *f*_RF2_, *f*_RF3_, -) are coupled into different probe waveguide units, from which all the scattering probe light interferes with the local light in the same bus waveguide. The multiplexed signals can be independently extracted by switching the down-conversion frequency *f*_LO_. This concept combined with surface functionalizations in different frequency channels may serve as an alternative approach for detecting several target analytes at the same time, which is highly desirable for practical applications such as medical diagnostics. In addition, we experimentally demonstrate that the probe-waveguide array can enhance the nanoparticle capture efficiency. As shown in Fig. 5c, representative beat intensity *I*_beat_ for the nine-waveguide (one-waveguide) configurations contains 21 (4) discrete steps induced by nanobead binding in 20 s. For the long-term monitoring over 450 s, the statistical analyses of time intervals between sequential nanobead-binding events are shown in Fig. 5d. The 1/*e* decay time is 0.95 s (2.96 s) for the nine-waveguide (one-waveguide) configurations, which indicates a 3-fold enhancement in capture efficiency.

### Discussion

We have demonstrated a 1/*f*-noise-free sensing scheme with an integrated heterodyne interferometer. Compared with the conventional lock-in amplifying technique, the sampling noise is suppressed by two orders of magnitude, pushing the single-particle detection limit down to the attogram level. The proposed scheme with ultra-low noise and high-gain signal amplification could significantly enhance the sensitivity and lower the detection limit, which gives an insight into the low-frequency dynamics of unlabeled nano-objects, such as antigen–antibody reactions and cell motions. Note that, with the low-frequency 1/*f* noise suppressed, the detection limit of the heterodyne interferometer is determined by the optical background noises in the probe and local light. Especially, the intensity of background probe light scattered due to the sidewall roughness of waveguides, which could be shifted beyond 1/*f* noise band with the signal and thus cannot be removed by the 1/*f*-noise-free scheme, hinders its ability operated at a precision limited by fundamental shot and thermal noises.

Since the 1/*f*-noise-free sensing scheme is compatible with previous enhancement mechanisms based on plasmonic nanostructures^15,17,31^ or surface charges^19,30^ on the target nanoparticles/molecules, the ultra-low noise optical sensor has a high potential for single-molecule dynamic detection. Furthermore, the CMOS-compatible heterodyne interferometer combined with the microfluidics and the chemical-functionalization technology could be widely applied in the fields of biology, medicine, pharmacology, physics, and environmental science.

## Methods

### Fabrication of on-chip waveguide sensor

The waveguide sensor is fabricated through a standard CMOS-compatible process on a silicon-on-insulator (SOI) 8" wafer with a 220-nm-thick top silicon layer and a 2-μm-thick buried oxide layer. The layout is patterned through deep ultraviolet lithography and inductively coupled plasma etching. All the grating couplers involved are designed for inducing the transverse electric (TE) field in waveguides, of which the measured insertion loss is about 7.5 dB/facet. We design and fabricate two kinds of sensing structures around the joint sensing area. For the first kind, the probe waveguides are sparsely arranged with a center pith of 50 μm, and each one together with the local waveguide form an independent sensing area. This structure is used to characterize the local image of the sensing region and the detection limit of the waveguide heterodyne sensing system. For the other kind, nine probe-waveguide and one local-waveguide are integrated to construct the joint sensing area. These probe waveguides with a center pitch of 5 μm are connected in parallel by a power splitter to form a dense array. The designed widths of the probe waveguide and local wavguide around joint sensing area are 1 μm and 500 nm, respectively (Fig. 5, insets).

### Experimental set-up

The wavelength of the laser source is 1550 nm. The AOM (Gooch *&* Housego Inc., PM FIBER-Q) is driven by an arbitrary function generator with a sinusoidal wave. The 1/*f*-noise-free signal extracting system consists of a high-pass filters (55 MHz), a BPD (Torlabs Inc., PDB570C), two low-noise photodetectors (Newport Inc. 1811-FC), a radio-frequency amplifier (Mini-circuits Inc., ZHL-1-2W-S+), a 90^∘^ power splitter centered at 80.18 MHz, two microwave mixers (Mini-circuits Inc., ZFM-2-S+), and a data acquisition system (National Instruments). The 2-way downcoverted signals (${A}_{{\rm{beat}}}^{{\rm{I}}}$ and ${A}_{{\rm{beat}}}^{{\rm{Q}}}$), as well as 1% power of the probe field and 10% power of the signal field for normalization are synchronously sampled by the DAQ.

### Sening characterization

In the experiment of nanotip scanning around joint sensing area, we use the one-probe-waveguide structure with a 1-μm-width gap. The nanotip is fabricated from a silica fiber through thermal-pulling (CO_2_ laser) and buffered hydrofluoric acid solution etching. The glass nozzle with an inner radius of 50 μm is fabricated by thermal-pulling the capillary glass tube (inner radius, 0.5 mm) using the hydrogen flame. The 3-axis translation stage drives the nanotip under the velocities of 0.45 μm/s (Fig. 2a) and 0.6 μm/s (Fig. 2b). Single nanobeads and HIV-1 VLPs are first diluted to tens of picomoles in the DI water (QDSphere Inc., radius of 30 nm; Nano-Micro Inc., radius of 41 nm; Invitrogen Inc., radius of 55 nm and HIV virus-like particles, radius of ~50 nm). The air flow with nanoparticles are then generated through the ultrasonic atomizer. The nanoparticles are deposited to the joint sensing area by a syringe pump (Harvard, Model PHD22/2000) at the flow rates of 15 ml min^−1^ for nanobeads and 10 ml min^−1^ for virus-like particles.

In the nanoparticles detection, the polystyrene nanobeads are measured using the waveguide sensor with a gap of 0.4, 1, and 1.4 μm for reducing the possible size correlations between the nanoparticles and the gap width. The step-finding algorithm is applied to extract the step signal. The step heights of the binding events are derived follows three steps: 1. Calculating the *δI*_beat_ through cross-correlation method from *I*_beat_ to identify the locations of the possible binding events. 2. Calculating all the possible step heights and recording the corresponding time. 3. Comparing all the step heights with the system noise level and preserving those that are greater than ~ 3*σ*. The total numbers of the deduced scattering events are 186 for the 30 nm nanoparticles, 195 for the 41 nm nanoparticles, and 156 for the 55 nm nanoparticles (Fig. 3).

### Cell culture and plasmid transfection

HEK293T cells (American Type Culture Collection) were cultured in Dulbecco’s Modified Eagle’s Medium (DMEM, Mediatech), supplemented with 10% (v/v) FBS (PANTM Biotech), 1 × GlutaMAXTM (Thermo Fisher) at 37 °C, 5% (v/v) CO_2_, and 90% relative humidity. Cells reaching 50–70% confluency in a T-75 tissue culture flask were transfected with 10 μg of the CMV-driven expression plasmid encoding HIV-1 Gag (pCR3.1-Gag, a kind gift of Dr. Sanford Simons at the Rockefeller University, New York, NY) using FuGENE^®^ 6 (Promega) as per manufacturer’s protocols.

### Virus particle collection

Virus-like particles were collected as previously described^35,36^. Briefly, culture supernatant of HEK293T cells harvested at 24 h after transfection of viral constructs was centrifuged at 1000 *g* for 10 min, followed by removal of cell debris and large aggregates with a 0.45 μm filter.

## Supplementary information

Supplementary Information

## Data Availability

The data that support the plots within this paper and other findings of this study are available from the corresponding author upon reasonable request. Source data for Figs. 1–5 are available at 10.6084/m9.figshare.13643729.

## Code availability

The codes that support the findings of this study are available from the corresponding authors upon reasonable request.

## References

[1] Hooge FN, Kleinpenning TGM, Vandamme LKJ (1981). Experimental studies on 1/*f* noise. Rep. Prog. Phys..

[2] Van der Ziel A (1988). Unified presentation of 1/*f* noise in electron devices: fundamental 1/*f* noise sources. Proc. IEEE.

[3] McDowell EJ, Cui X, Yaqoob Z, Yang C (2007). A generalized noise variance analysis model and its application to the characterization of 1/*f* noise. Opt. Express.

[4] Balandin AA (2013). Low-frequency 1/*f* noise in graphene devices. Nat. Nanotechnol..

[5] Dutta P, Horn PM (1981). Low-frequency fluctuations in solids: *1/f* noise. Rev. Mod. Phys..

[6] DeFelice LJ (1976). 1/*f* resistor noise. J. Appl. Phys..

[7] Motchenbacher, C. D. & Connelly, J. A. *Low Noise Electronic System Design* (Wiley, 1993).

[8] Baaske MD, Vollmer F (2016). Optical observation of single atomic ions interacting with plasmonic nanorods in aqueous solution. Nat. Photon..

[9] Vollmer F, Arnold S, Keng D (2008). Single virus detection from the reactive shift of a whispering-gallery mode. Proc. Natl Acad. Sci. USA.

[10] Shao L (2013). Detection of single nanoparticles and lentiviruses using microcavity resonance broadening. Adv. Mater..

[11] Yu XC (2014). Single nanoparticle detection and sizing using a nanofiber pair in an aqueous environment. Adv. Mater..

[12] Yu X-C (2018). Optically sizing single atmospheric particulates with a 10-nm resolution using a strong evanescent field. Light Sci. Appl..

[13] Tang SJ (2018). On-chip spiral waveguides for ultrasensitive and rapid detection of nanoscale objects. Adv. Mater..

[14] Mauranyapin NP (2019). Quantum noise limited nanoparticle detection with exposed-core fiber. Opt. Express.

[15] Li H (2019). Single-molecule detection of biomarker and localized cellular photothermal therapy using an optical microfiber with nanointerface. Sci. Adv..

[16] Kitamura K, Tokunaga M, Iwane AH, Yanagida T (1999). A single myosin head moves along an actin filament with regular steps of 5.3 nanometres. Nature.

[17] Baaske MD, Foreman MR, Vollmer F (2014). Single-molecule nucleic acid interactions monitored on a label-free microcavity biosensor platform. Nat. Nanotechnol..

[18] Kim E, Baaske MD, Schuldes I, Wilsch PS, Vollmer F (2017). Label-free optical detection of single enzyme-reactant reactions and associated conformational changes. Sci. Adv..

[19] Mauranyapin NP, Madsen LS, Taylor MA, Waleed M, Bowen WP (2017). Evanescent single-molecule biosensing with quantum-limited precision. Nat. Photon..

[20] Foreman MR, Jin W-L, Vollmer F (2014). Optimizing detection limits in whispering gallery mode biosensing. Opt. Express.

[21] Freudiger CW (2014). Stimulated raman scattering microscopy with a robust fibre laser source. Nat. Photon..

[22] Cao Z (2019). Biochemical sensing in graphene-enhanced microfiber resonators with individual molecule sensitivity and selectivity. Light Sci. Appl..

[23] He LN, Ozdemir K, Zhu JG, Kim W, Yang L (2011). Detecting single viruses and nanoparticles using whispering gallery microlasers. Nat. Nanotechnol..

[24] Ozdemir SK (2014). Highly sensitive detection of nanoparticles with a self-referenced and self-heterodyned whispering-gallery raman microlaser. Proc. Natl Acad. Sci. USA.

[25] Li BB (2014). Single nanoparticle detection using split-mode microcavity raman lasers. Proc. Natl Acad. Sci. USA.

[26] Zhu J (2009). On-chip single nanoparticle detection and sizing by mode splitting in an ultrahigh-Q microresonator. Nat. Photon..

[27] Knittel J, Swaim JD, McAuslan DL, Brawley GA, Bowen WP (2013). Back-scatter based whispering gallery mode sensing. Sci. Rep..

[28] Su J, Goldberg AF, Stoltz BM (2016). Label-free detection of single nanoparticles and biological molecules using microtoroid optical resonators. Light Sci. Appl..

[29] Lu T (2011). High sensitivity nanoparticle detection using optical microcavities. Proc. Natl Acad. Sci. USA.

[30] Vincent S, Subramanian S, Vollmer F (2020). Optoplasmonic characterisation of reversible disulfide interactions at single thiol sites in the attomolar regime. Nat. Commun..

[31] Lee I-H, Yoo D, Avouris P, Low T, Oh S-H (2019). Graphene acoustic plasmon resonator for ultrasensitive infrared spectroscopy. Nat. Nanotechnol..

[32] Faez S (2015). Fast, label-free tracking of single viruses and weakly scattering nanoparticles in a nanofluidic optical fiber. ACS Nano.

[33] Petersen J, Volz J, Rauschenbeutel A (2014). Chiral nanophotonic waveguide interface based on spin-orbit interaction of light. Science.

[34] Herfst S (2012). Airborne transmission of influenza A/H5N1 virus between ferrets. Science.

[35] Qu N (2018). Inhibition of retroviral Gag assembly by non-silencing miRNAs promotes autophagic viral degradation. Protein Cell.

[36] Yang Y (2018). Roles of Gag-RNA interactions in HIV-1 virus assembly deciphered by single-molecule localization microscopy. Proc. Natl Acad. Sci. USA.

