# Supplementary material for: 1/f-noise-free optical sensing with an integrated heterodyne interferometer
Source: Nat Commun. 2021 Mar 30;12:1973. doi: 10.1038/s41467-021-22271-4 (PMC8009908; doi:10.1038/s41467-021-22271-4)
Supplement: Supplementary file 1 — Supplementary Information [file 41467_2021_22271_MOESM1_ESM.pdf]

Supplementary Information for

1/*f*-noise-free optical sensing with an integrated heterodyne interferometer

Ming Jin[1,7], Shui-Jing Tang[2,7], Jin-Hui Chen[2], Xiao-Chong Yu[2], Haowen Shu[1], Yuansheng Tao[1], Antony K. Chen[3], Qihuang Gong[2,4,5,6], Xingjun Wang[1,4,6,†] and Yun-Feng Xiao[2,4,5,6,‡]

[1]State Key Laboratory of Advanced Optical Communications System and Networks, Department of Electronics, School of Electronics Engineering and Computer Science, Peking University, Beijing, 100871, China.

[2]State Key Laboratory for Artificial Microstructure and Mesoscopic Physics, School of Physics, Peking University, Beijing 100871, China.

[3]Department of Biomedical Engineering, College of Engineering, Peking University, Beijing 100871, China.

[4]Frontiers Science Center for Nano-optoelectronics, Peking University, Beijing 100871, China.

[5]Collaborative Innovation Center of Extreme Optics, Shanxi University, Taiyuan 030006, China.

[6]Peking University Yangtze Delta Institute of Optoelectronics, Nantong 226010, China.

[7]These authors contributed equally.

Corresponding authors: †xjwang@pku.edu.cn, ‡yfxiao@pku.edu.cn.

Supplementary Figures

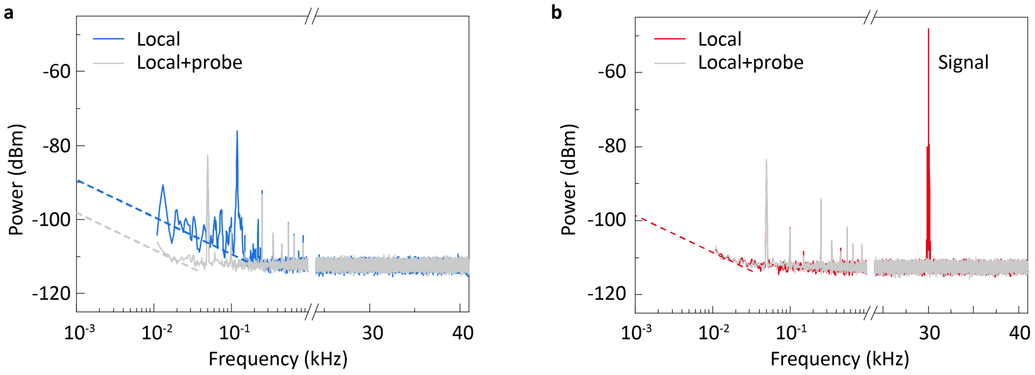

Supplementary Fig. 1: Background noise. Power spectra of $A^{\mathrm{I}}_{\mathrm{beat}}$ at low frequency region when probe light are off (gray curves) or on (light curves) at different bias frequencies. **a**, Conventional lock-in method ($\Delta f = 0$ Hz). **b**, $1/f$-noise-free scheme ($\Delta f =$ 30 kHz). The low-frequency noise floor is fitted by $1/f$ (dashed curves). Here, the signal peak in **a** is not presented due to the bandwidth limitation of the electrical spectrum analyzer.

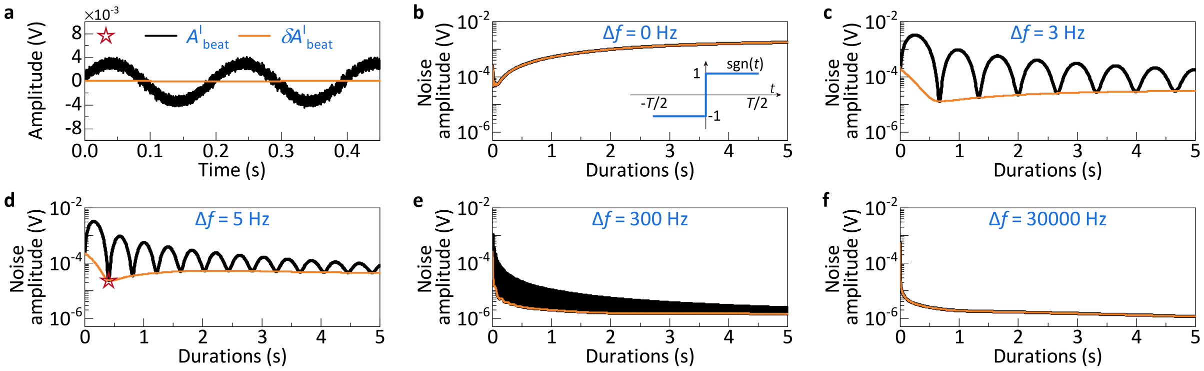

Supplementary Fig. 2: Sampling noise analysis. a, Sampled beat amplitude $A^{\mathrm{I}}_{\mathrm{beat}}$ under the bias frequency of $\Delta f = 5$ Hz and the deduced amplitude change $\delta A^{\mathrm{I}}_{\mathrm{beat}}$ over the duration of $T = 0.405$ s. The calculated noise amplitude is labeled in d with the pentagram. **b-f**, the derived noise amplitude versus the durations under different bias frequencies Δf.

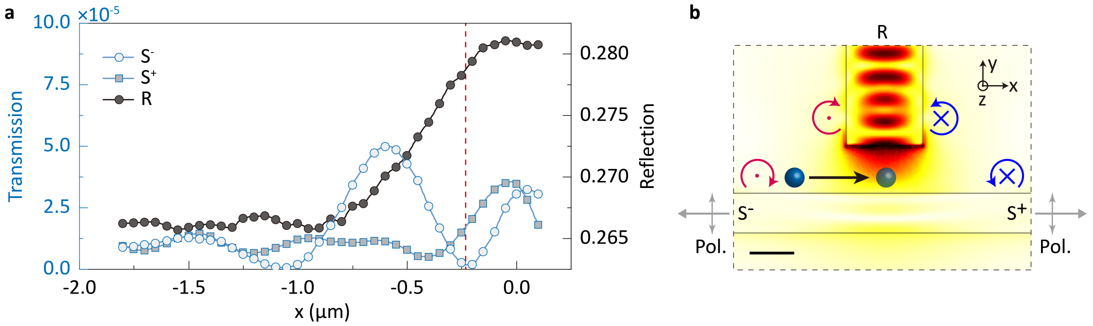

Supplementary Fig. 3: Nanoparticle induced directional scattering. a, Calculated transmission at different output ports. Blue line with circles (squares), transmission of the probe light at output port $S^-(S^+)$ when the nanotip is scanned through the joint sensing region; black line with circles, reflection of the scattering light at probe waveguide input port R; red dashed line, the position ($x = -0.25$ μm) in x-axis of the 3D FEM model corresponding to the zero-coordinate position in Fig. 3a. **b,** Waveguide mode distribution and nanotip scan range in the simulation. Red arrows and blue arrows represent electric field. Dots and crosses indicate the spin angular momentum of the light. Gray arrows represent the output light directions and corresponding polarizations at each port. Scale bar: 500 nm.

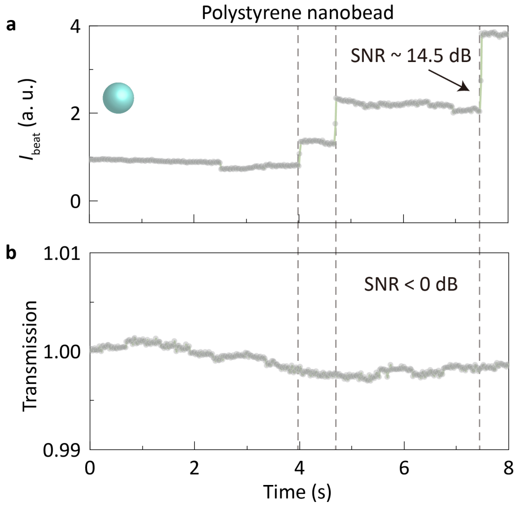

Supplementary Fig. 4: Sensing performance comparison. Beat intensity I_{beat} (a) and transmitted power of local waveguide (b) when single polystyrene nanobeads with the radius of 30 nm are deposited at the joint sensing region.

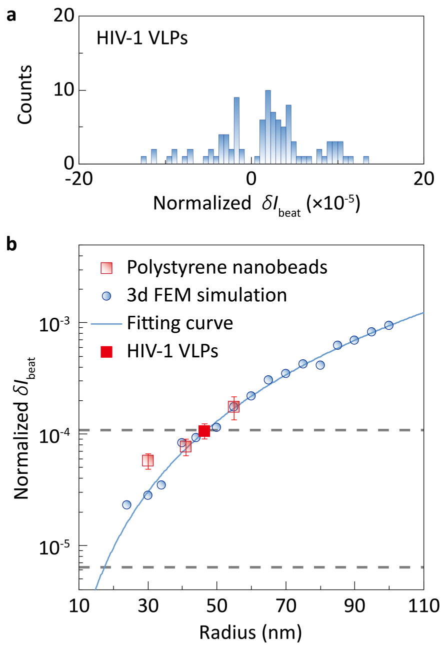

Supplementary Fig. 5: HIV-1 VLPs sizing. a, Histograms of normalized beat intensity change δI_{beat} for HIV-1 VLPs. **b**, The purple square is the normalized δI_{beat} induced by HIV-1 virus-like particles.

Supplementary Notes

Supplementary note 1: Theoretical description of dark-field heterodyne interferometer operating at the $1/f$-noise-free regime.

When a nanoparticle is deposited onto the joint sensing area in Fig. 1b, it scatters the probe light. Due to the fact that particle radius (below 100 nm) is much smaller than the optical wavelength (λ), the scattering field could be described by the dipole scattering, and its dipole scattering cross-section is

$$\sigma_{\mathrm{p}} = \frac{8k^4a^4}{3}\left(\frac{\varepsilon_{\mathrm{p}}/\varepsilon_{\mathrm{m}}-1}{\varepsilon_{\mathrm{p}}/\varepsilon_{\mathrm{m}}+2}\right)\pi a^2, \tag{1}$$

where $k = 2\pi/\lambda$ is the wave number, a is the radius of the nanosphere, ε_{p} and ε_{m} are the permittivity of the particle and the surrounding medium, respectively. This scattering probe light is collected by the local waveguide with the efficiency of $\eta_{\mathrm{c}} \sim f(a, \boldsymbol{r})$, which is a function of the radius and location of particle. This relation is influenced by the overlap of local light field and the dipole field. Therefore, the collecting efficiency can be described as

$$\eta_{\mathrm{s}} \propto \eta_{\mathrm{c}}\frac{8k^4a^4}{3}\left(\frac{\varepsilon_{\mathrm{p}}/\varepsilon_{\mathrm{m}}-1}{\varepsilon_{\mathrm{p}}/\varepsilon_{\mathrm{m}}+2}\right)^2\pi a^2. \tag{2}$$

Then, the collected probe light with a power of $P_{\mathrm{col}} = E_{\mathrm{probe}}^2\eta_{\mathrm{s}}$ interferes with the local light, the combined light field is

$$E_{\mathrm{beat}} = E_{\mathrm{probe}}\sqrt{\eta_{\mathrm{s}}}e^{i(\omega_{\mathrm{probe}}t+\phi_{\mathrm{probe}})} + E_{\mathrm{local}}e^{i(\omega_{\mathrm{local}}t+\phi_{\mathrm{local}})}, \tag{3}$$

where the subscripts j = probe or local, indicate the probe and local light with electrical amplitude E_j, ω_j and ϕ_j are the angular frequency and optical phase, respectively. In our system, the frequency difference between the probe and the local light is $f_{\mathrm{RF}} = (\omega_{\mathrm{probe}} - \omega_{\mathrm{local}})/2\pi = 80.15$ MHz. The combined light is detected by a BPD together with a reference light (E_{ref}). The electrical responses of the BPD are

$$\begin{aligned} i_{\mathrm{BPD1}} &= R_{\mathrm{BPD}} \cdot E_{\mathrm{beat}} \cdot E_{\mathrm{beat}}^{*} \\ &= R_{\mathrm{BPD}}\left[I_{\mathrm{probe}}\eta_{\mathrm{s}} + I_{\mathrm{local}} + 2\cdot\sqrt{I_{\mathrm{probe}}\eta_{\mathrm{s}}I_{\mathrm{local}}}\cdot\cos(2\pi f_{\mathrm{RF}}t+\Delta\phi)\right], \end{aligned} \tag{4}$$

$$i_{\mathrm{BPD2}} = R_{\mathrm{BPD}} \cdot E_{\mathrm{ref}} \cdot E_{\mathrm{ref}^{*}}, \tag{5}$$

$$\begin{aligned} i_{\mathrm{BPD}} &= i_{\mathrm{BPD1}} - i_{\mathrm{BPD2}} \\ &= R_{\mathrm{BPD}}\left[I_{\mathrm{probe}}\eta_{\mathrm{s}} + I_{\mathrm{local}} - I_{\mathrm{ref}} + 2\cdot\sqrt{I_{\mathrm{probe}}\eta_{\mathrm{s}}I_{\mathrm{local}}}\cdot\cos(2\pi f_{\mathrm{RF}}t+\Delta\phi)\right], \end{aligned} \tag{6}$$

where i_{BPD1} and i_{BPD2} represent the photocurrents of the two detectors of the BPD, R_{BPD} is the responsivity of the commercial photodiode, $\Delta\phi = \phi_{\text{probe}} - \phi_{\text{local}}$ is the phase difference between the probe and local light field, E_{ref} is the amplitude of the reference light. Compared with the direct monitoring the transmission of scattering probe light ($I_{\text{probe}}\eta_{\text{s}}$), the desired amplitude change ($2 \cdot \sqrt{I_{\text{probe}}\eta_{\text{s}}I_{\text{local}}}$) of the beat note has an enhancement factor of $2 \cdot \sqrt{I_{\text{local}}/I_{\text{probe}}\eta_{\text{s}}}$ under the heterodyne concept. Here, to eliminate the laser common-mode noise[1], the power of the reference light is set with $I_{\text{probe}}\eta_{\text{s}} + I_{\text{local}} = I_{\text{ref}}$. Therefore, the output is

$$v_{\text{BPD}} = i_{\text{BPD}}z = 2z \cdot R_{\text{BPD}}\sqrt{I_{\text{probe}}\eta_{\text{s}}I_{\text{local}}} \cdot \cos(2\pi f_{\text{RF}}t + \Delta\phi), \tag{7}$$

where z is the impedance of the BPD, v_{BPD} is output voltage of signal. Since it is hard to keep a perfect balance between the two arms of BPD, a high-pass filter is introduced to remove the direct current. Such RF signal is boosted by an electrical amplifier and followed by a high-pass filter for further noise suppression. The beat signal after amplifying and filtering is

$$v_{\text{BPD}} = 2z \cdot 10^{(G_{\text{amp.}} - L_{\text{filter}})/10} \cdot R_{\text{BPD}}\sqrt{I_{\text{probe}}\eta_{\text{s}}I_{\text{local}}} \cdot \cos(2\pi f_{\text{RF}}t + \Delta\phi). \tag{8}$$

From the handbooks provided by the amplifier and the filter (electrical gain of the amplifier: $G_{\text{amp.}} = 29$ dB, the loss of filter at 80 MHz: $L_{\text{filter}} \sim 0.5$ dB), this process brings an amplification of about 700 times to the signal. Then, this amplified signal is recorded through the cross-phase sampling scheme. The signal passes through power splitter to separate two RF signals with equal power:

$$v_{\text{beat}}^{\text{I}} = v_{\text{beat}}^{\text{Q}} = \sqrt{2}z \cdot 10^{(G_{\text{amp.}} - L_{\text{filter}})/10} \cdot R_{\text{BPD}}\sqrt{I_{\text{probe}}\eta_{\text{s}}I_{\text{local}}} \cdot \cos(2\pi f_{\text{RF}}t + \Delta\phi). \tag{9}$$

This beat signal is transferred to the sampling carrier with a bias frequency $\Delta f = f_{\text{LO}} - f_{\text{RF}} = 30$ kHz. which is well beneath the sampling threshold (50 kHz) of the DAQ system. Before down converting, electrical LO is divided by a 90° power splitter with equal power but orthogonal phase:

$$v_{\text{ELO1}} = A_{\text{ELO}}\cos(2\pi f_{\text{ELO}}t + \phi_{\text{ELO}}), \tag{10a}$$

$$v_{\text{ELO2}} = A_{\text{ELO}}\sin(2\pi f_{\text{ELO}}t + \phi_{\text{ELO}}). \tag{10b}$$

They are separately mixed with the I/Q signal, and the output beat signals are finally

recorded as the original data:

$$v_{\text{mixing}}^{\text{I}} = v_{\text{ELO1}} \cdot v_{\text{beat}}^{\text{I}} = A_{\text{E}}\sqrt{I_{\text{probe}}\eta_{\text{s}}I_{\text{local}}}\left(\cos\alpha_{+} + \cos\alpha_{-}\right), \tag{11a}$$

$$v_{\text{mixing}}^{\text{Q}} = v_{\text{ELO1}} \cdot v_{\text{beat}}^{\text{Q}} = A_{\text{E}}\sqrt{I_{\text{probe}}\eta_{\text{s}}I_{\text{local}}}\left(\sin\alpha_{+} - \sin\alpha_{-}\right), \tag{11b}$$

where $\alpha_{+} = (f_{\text{ELO}} + f_{\text{RF}})\,t + (\phi_{\text{ELO}} + \Delta\phi)$, $\alpha_{-} = (f_{\text{ELO}} - f_{\text{RF}})\,t + (\phi_{\text{ELO}} - \Delta\phi)$ are the sum and difference frequency components, respectively. $A_{\text{E}} = \frac{\sqrt{2}}{2} z A_{\text{ELO}} \cdot 10^{(G_{\text{amp}} - L_{\text{filter}})/10}$ is the equipment-dependent scale factor. After low frequency filtering, the sum-frequency components are filtered, and only the beat amplitude changes with the bias frequency $\Delta f =$ 30 kHz are sampled as

$$A_{\text{beat}}^{\text{I}} = A_{\text{s}} \cdot \cos\alpha_{-}, \tag{12a}$$

$$A_{\text{beat}}^{\text{Q}} = A_{\text{s}} \cdot \sin\alpha_{-}. \tag{12b}$$

Where $A_{\text{s}} = A_{\text{E}}\sqrt{I_{\text{probe}}\eta_{\text{s}}I_{\text{local}}}$ contains the signal information. The sampled noise amplitude of this two-way orthogonal signal is estimated in Fig. 1d and Supplementary Fig. 2.

To extract the envelope of the beat note, several digital processing procedures are applied. First, the beat intensity I_{beat} is recovered from the sampled $A_{\text{beat}}^{\text{I}}$ and $A_{\text{beat}}^{\text{I}}$ by,

$$I_{\text{beat}} = (A_{\text{beat}}^{\text{I}})^2 + (A_{\text{beat}}^{\text{Q}})^2 = A_{\text{s}}^2 = A_{\text{E}}^2 I_{\text{probe}}\eta_{\text{s}}I_{\text{local}}. \tag{13}$$

Here, A_{E} remains the same in the experiments. On the contrary, I_{local} and I_{probe} vary from different measurements. Therefore, we track 10% of the output signal light and 1% of the input probe light simultaneously with beat signal for normalization. Finally, an average filtering method is applied to further eliminate the noise with the frequency beyond 50 Hz.

Supplementary note 2: Calculation of sampling noise amplitude

We perform a sequential measurement to reveal the influence of the bias frequency Δf on the real-time amplitude fluctuations of the raw signal. To evaluate noise performance of this sampled raw signal $A^{\mathrm{I}}_{\mathrm{beat}}$, the noise amplitude defined by the standard deviation of the amplitude change $\delta A^{\mathrm{I}}_{\mathrm{beat}}$ is used,

$$\delta A^{\mathrm{I}}_{\mathrm{beat}} = \frac{2}{T}\int_{-T/2}^{T/2} A^{\mathrm{I}}_{\mathrm{beat}}(t+\tau)h(\tau)d\tau, \tag{14}$$

where $h(t) = sgn(t)$ and T is the duration time. According to Supplementary Equation (12), the sampled $A^{\mathrm{I}}_{\mathrm{beat}}$ is a radio-frequency signal with the frequency of Δf, as shown in Supplementary Fig. 2a (black curve). Therefore, the standard deviation of the amplitude change shows an oscillation characteristic over the duration T as shown (e.g., Supplementary Fig. 2d, black curves). It is found that, the minima of the oscillating noise amplitude are free from such influence. For example, for the minimal value labelled by the pentagram in Supplementary Fig. 2d, corresponding $\delta A^{\mathrm{I}}_{\mathrm{beat}}$ is shown in Supplementary Fig. 2a (orange curve) behaving no significant periodic fluctuations. Therefore, the sampling noise amplitude (Supplementary Figs. 2b-f, orange curves) under different bias frequency Δf over the durations T are derived from the interpolation of the corresponding minima in black curves (Supplementary Figs. 2b-f). The sampling noise amplitude at T= 1 s is plotted in Fig. 1d, which illustrates an relation of $1/\Delta f^{\gamma}$ (γ= 0.61) between the noise amplitude and the bias frequency Δf.

Supplementary note 3: Nanoparticle induced chiral mode scattering

When a nanoparticle is attached to the joint sensing region, the total power of the light scattered into the local waveguide mode is

$$I_{\text{sca}} \propto |\mathbf{d}^* \cdot \boldsymbol{\epsilon}(\mathbf{r})|^2 = |\alpha^* \boldsymbol{\varepsilon}^*_{\text{exc}}(\mathbf{r}) \cdot \boldsymbol{\epsilon}(\mathbf{r})|^2, \tag{15}$$

where $\mathbf{d}$ is the induced dipole moment of a spherical scatter, α is the complex polarizability, $\boldsymbol{\varepsilon}_{\text{exc}}(\mathbf{r})$ is the illuminated light field from the probe waveguide, and $\boldsymbol{\epsilon}(\mathbf{r})$ is the electric field of the guided mode in the local waveguide. As a result, the collecting efficiency of the local waveguide is proportional to the overlap between the evanescent fields of the probe and local guided modes at the particle's position[2]. Due to the strong transverse confinement of nanowaveguide, the evanescent field has a longitudinal polarization component in the propagation direction of the light. As shown in Supplementary Fig. 3b, the evanescent fields around the probe and local waveguides in the joint sensing area are $\mathbf{E}_x - i\mathbf{E}_y$, $-i\mathbf{E}'_x - \mathbf{E}'_y$ (the direction of S^-) and $i\mathbf{E}'_x - \mathbf{E}'_y$ (the direction of S^+), respectively. The corresponding power of the probe light scattered by nanoparticle into S^- and S^+ are

$$I^{S-}_{\text{sca}} \propto |(\mathbf{E}_x - i\mathbf{E}_y)^* \cdot (-i\mathbf{E}'_x - \mathbf{E}'_y)|^2 = |E_x E'_x + E_y E'_y|^2, \tag{16}$$

$$I^{S+}_{\text{sca}} \propto |(\mathbf{E}_x - i\mathbf{E}_y)^* \cdot (i\mathbf{E}'_x - \mathbf{E}'_y)|^2 = |E_x E'_x - E_y E'_y|^2. \tag{17}$$

Therefore, the scattering power of the probe light at different ports of the local waveguide is asymmetric and shows a position-dependent behaviour. To quantitatively calculate the scattering efficiency of a nano-object, we performed the 3D FEM simulation, and both the inhomogeneous light field and interface interactions are included (Supplemeantary Fig. 3). When a nano-object is deposited at the center ($x = 0$) of the joint sensing area, the quasi-linear polarized light scattered from the the probe waveguide interacts with the interface chiral field of the local waveguide, which leads to equally scattering efficiency at the forward (S^+) and backward (S^-) direction. When a nano-object is close to left edge of probe waveguide ($x \sim 0.5$ µm), the constructive chiral field interaction between the scattered light and the local waveguide mode leads to strong backward coupling (S^-). Considering the diffraction effect of probe light field, the maximum backward coupling (S^-) position is slightly deviated from the 0.5 µm. We find that the reflection effect at the joint sensing region also influences the scattering efficiency as shown in black line in Supplementary Fig. 3. To reduce the fluctuations of the probe light power, an optical isolator is required at

the probe waveguide input (not drawn in Fig. 1b). Note that, during the nanotip scanning experiment, the position of the nanotip in the main text is figured out through an optical microscope. Considering radius of nanotip ($\sim$ 200 nm) and the angle between the nanotip and the chip surface, the transmission of the scattering probe light at the S^- port within the range of $x \in [-1.75, -0.25]$ (µm) is used to fit the experimental results.

[1] Freudiger, C. W. *et al.* Stimulated raman scattering microscopy with a robust fibre laser source. *Nat. Photon.* **8**, 153 (2014).

[2] Petersen, J., Volz, J. & Rauschenbeutel, A. Chiral nanophotonic waveguide interface based on spin-orbit interaction of light. *Science* **346**, 67 (2014).
